# 880. Diagnostic Testing Sample-Type Preferences: Results From An International Survey On SARS-CoV-2

**DOI:** 10.1093/ofid/ofad500.925

**Published:** 2023-11-27

**Authors:** Sumaira Akbarzada, Nithya Narayanan, Leah Salzano, Emily R Tobik, Sarah Megiel, Craig Duni, Brittany Choate, Samantha S Harper, Anne L Wyllie

**Affiliations:** Yale University, New Haven, Connecticut; Yale University, New Haven, Connecticut; Yale School of Public Health, New Haven, Connecticut; Yale University, New Haven, Connecticut; Yale University, New Haven, Connecticut; Yale University, New Haven, Connecticut; Yale University, New Haven, Connecticut; Yale University, New Haven, Connecticut; Yale School of Public Health, New Haven, Connecticut

## Abstract

**Background:**

Diagnostic strategies rapidly evolved during the COVID-19 pandemic in response to the unprecedented worldwide testing demand. The public’s perception in regards to testing has also evolved. Testing remains essential for understanding the current state of the pandemic and directing efforts and resources. The purpose of this study was to better understand the public view regarding clinical sample types to inform future diagnostics development and surveillance planning.

**Methods:**

To assess SARS-CoV-2 testing preferences, we distributed a Qualtrics survey via social media and email to connections in > 100 countries. The questionnaire covered testing preferences and key demographic information. Python was used to analyze the raw data.

**Results:**

From 03/30-11/30/2022, a total of 2,094 responses were received from Africa (22%), Asia (8%), Europe (22%), North America (27%), Latin America and the Caribbean (9%), the Middle East (7%), and Oceania (6%). Participants were 55% female, 44% male, and 1% non-binary and ranged in ages (18-24, 13%; 25-34, 27%; 35-44, 23%; 45-54, 16%; 55-64, 14%; 65+, 8%). Education level and employment was skewed with 52% holding a graduate degree and 26% holding a bachelor’s degree; 27% were scientists/researchers and 29% were healthcare workers. Assuming equal accuracy between COVID-19 test types, respondents most preferred oral sample collection and least preferred methods involving coughing. The most preferred sample collection method varied by region (nasal swab = Europe; oral swab = Africa, North America, Oceania; saliva = Asia, Latin America and the Caribbean). In the majority of regions, saliva based testing was the most preferred method by parents and guardians for their children.

**Conclusion:**

This study identified a preference for oral sample types for the detection of SARS-CoV-2, with saliva collection from children preferred by parents. Large-scale, easily accessible diagnostic testing is vital for detecting, monitoring, and containing outbreaks of respiratory pathogens; utilization of oral sample types supports the feasibility of this, if individuals are then more likely to test. Results from this study should be considered when new testing practices are designed to encourage maximum participation from individuals and improve community health.

**Disclosures:**

**Anne L. Wyllie, PhD**, Co-Diagnostics: Board Member|Merck: Grant/Research Support|Pfizer: Advisor/Consultant|Pfizer: Grant/Research Support

